# Characterizing a complex CT-rich haplotype in intron 4 of *SNCA* using large-scale targeted amplicon long-read sequencing

**DOI:** 10.1038/s41531-024-00749-4

**Published:** 2024-07-26

**Authors:** Pilar Alvarez Jerez, Kensuke Daida, Francis P. Grenn, Laksh Malik, Abigail Miano-Burkhardt, Mary B. Makarious, Jinhui Ding, J. Raphael Gibbs, Anni Moore, Xylena Reed, Mike A. Nalls, Syed Shah, Medhat Mahmoud, Fritz J. Sedlazeck, Egor Dolzhenko, Morgan Park, Hirotaka Iwaki, Bradford Casey, Mina Ryten, Cornelis Blauwendraat, Andrew B. Singleton, Kimberley J. Billingsley

**Affiliations:** 1 Laboratory of Neurogenetics, National Institute on Aging, Bethesda, MD USA; 2 Center for Alzheimer’s and Related Dementias, National Institute on Aging, Bethesda, MD USA; 3 Department of Neurodegenerative Disease, UCL Queen Square Institute of Neurology, University College London, London, UK; 4 DataTecnica LLC, Washington, DC USA; 5 Human Genome Sequencing Center, Baylor College of Medicine, One Baylor Plaza, Houston, TX USA; 6 Department of Computer Science, Rice University, Houston, TX USA; 7 Pacific Biosciences, Menlo Park, CA USA; 8 NIH Intramural Sequencing Center, National Human Genome Research Institute, National Institutes of Health, Bethesda, MD USA; 9 The Michael J. Fox Foundation for Parkinson’s Research, New York, New York, USA; 10 Genetics and Genomic Medicine, Great Ormond Street Institute of Child Health, University College London, London, UK; 11 Uk Dementia Research Institute at the University of Cambridge and Department of Clinical Neurosciences, University of Cambridge, Cambridge Biomedical Campus, Cambridge, UK; 12 Aligning Science Across Parkinson’s (ASAP) Collaborative Research Network, Chevy Chase, MD USA

**Keywords:** Genomics, Next-generation sequencing

## Abstract

Parkinson’s disease (PD) is a common neurodegenerative disorder with a significant risk proportion driven by genetics. While much progress has been made, most of the heritability remains unknown. This is in-part because previous genetic studies have focused on the contribution of single nucleotide variants. More complex forms of variation, such as structural variants and tandem repeats, are already associated with several synucleinopathies. However, because more sophisticated sequencing methods are usually required to detect these regions, little is understood regarding their contribution to PD. One example is a polymorphic CT-rich region in intron 4 of the *SNCA* gene. This haplotype has been suggested to be associated with risk of Lewy Body (LB) pathology in Alzheimer’s Disease and *SNCA* gene expression, but is yet to be investigated in PD. Here, we attempt to resolve this CT-rich haplotype and investigate its role in PD. We performed targeted PacBio HiFi sequencing of the region in 1375 PD cases and 959 controls. We replicate the previously reported associations and a novel association between two PD risk SNVs (rs356182 and rs5019538) and haplotype 4, the largest haplotype. Through quantitative trait locus analyzes we identify a significant haplotype 4 association with alternative CAGE transcriptional start site usage, not leading to significant differential *SNCA* gene expression in post-mortem frontal cortex brain tissue. Therefore, disease association in this locus might not be biologically driven by this CT-rich repeat region. Our data demonstrates the complexity of this *SNCA* region and highlights that further follow up functional studies are warranted.

## Introduction

Parkinson’s Disease (PD) is a complex neurodegenerative disease presenting with tremor, muscle stiffness, dystonia, and impaired balance, among other symptoms. It is the second most common neurodegenerative disease after Alzheimer’s Disease, affecting approximately 1 million people in North America^1^. Within the Northern European ancestry population 3–5% of PD cases can be linked to known Parkinson’s disease causing mutations and 90 genetic risk variants collectively explain an estimated 16–36% of heritable risk^2,3^. Genetic variants in the *SNCA*, *LRRK2*, *VPS35*, *PRKN*, and *PINK1* genes, among others, have been associated with both autosomal dominant and recessive forms of the disease^4^.

Most genetic studies to date have focused on the role of single nucleotide variants (SNVs), but structural variants (SVs) can have a large impact due to their size and complexity. As such, there has been an increased focus on research regarding the role of SVs on disease^5–7^. However, due to their complexity, traditional second generation sequencing methods have not been able to accurately detect or resolve many SVs. Recent developments in both long-read sequencing technology and data analysis methods have created tools to reduce alignment error and subsequently improve variant calling in these areas of the genome^8^. New technologies such as HiFi (Pacific Biosciences), for example, have improved methods to address previous difficulties in calling SVs^9^.

The *SNCA* gene, located on chromosome 4, encodes for the alpha-synuclein protein and aggregates of this protein are found in Lewy bodies, a known pathological hallmark of PD^10^. Rare SVs across the *SNCA* locus have been shown as causative of autosomal dominant PD through increasing gene dosage^11,12^. It has been reported that a polymorphic CT-rich SV in intron 4 of *SNCA* is associated with risk of Lewy Body (LB) pathology in Alzheimer’s disease (AD) and affects *SNCA* expression^13^. Four different haplotypes were identified in this intronic region through Sanger sequencing in 95 AD and LB/AD samples. Additionally, real-time PCR was used to quantify human *SNCA-*mRNA levels in 334 AD and LB/AD samples where haplotype 3 was associated with higher levels of *SNCA*-mRNA expression. The four haplotypes were later confirmed in 12 human brain samples using PacBio SMRT sequencing^14^.

Here, we aimed to resolve the CT-rich haplotype in intron 4 of *SNCA* and investigate its role in PD. We performed targeted PacBio HiFi sequencing of the region in 1375 PD cases and 959 controls and leveraged existing expression datasets from the frontal cortex to perform quantitative trait locus (QTL) analyzes to investigate the functional consequence of this haplotype.

## Results

### Pacbio data overview

Here we investigate a complex haplotype within intron 4 of the *SNCA* gene in a large number of PD cases and controls. After quality control, 1842 samples remained including 1080 PD cases and 762 controls, with a median of 327 coverage of the region chr4:89821149-89821669. We next ran TRGT and binned our samples into the four haplotypes as described in the methods. After binning, the estimated allele frequency for each haplotype was in line with the original report^13^, for exact frequencies per cohort, see supplementary table 1. To confirm correct grouping of our haplotypes, we used an edit distance analysis to compare our haplotype sequences against the reported sequences. An edit distance analysis calculates the number of base pair differences between two sequences. No sample had an edit distance above one, meaning our haplotype sequences varied at most by one base pair compared to the reported haplotype sequences.

### The CT-rich *SNCA* haplotype is associated with known risk variants of Synucleinopathies

Next, to investigate whether known SNVs were tagging our haplotypes, we explored the relationship between our haplotypes and known PD (rs356182, rs5019538, rs2298728) and LBD (rs7680557) risk SNVs in *SNCA* identified by existing GWAS studies. Out of the 1842 samples remaining, 1601 had SNV information available for rs356182, rs5019538, rs2298728, and rs7680557 (928 cases and 673 controls) from Illumina short-read WGS. These 1601 samples were taken forward for further analysis. After controlling for sex, age, and genetic PCs 1–5 as covariates, we found a significant association between all four haplotypes and our four SNVs, with different directionalities using a linear regression. Haplotypes 2 and 3 were positively associated with rs7680557 (*p* = 1.64 × 10^-90^ and *p* = 6.56 × 10^-25^), the LBD SNV, which is in line with previous reports detailing the association of these two haplotypes with cases of Alzheimer’s Disease with Lewy bodies. Additionally, haplotype 3 was positively associated with rs2298728 (*p* = 2.88 × 10^-290^), as was previously reported, and with the rs356182 PD SNV (*p* = 2.78 × 10^-13^) and haplotype 4 was positively associated with both the rs356182 and rs5019538 PD SNVs (*p* = 4.53 × 10^-56^ and *p* = 1.86 × 10^-97^, respectively). Linkage disequilibrium (LD) analysis did not initially reveal a high LD pattern between haplotypes and SNVs, with the highest D’ = 0.76 between haplotype 3 and the rs2298728 SNV. However, when looking exclusively at homozygous carriers of each haplotype, we see that 90.50% and 96.30% of haplotype 2 and 3 homozygotes respectively carry the LBD rs7680557 SNV in at least one allele and 97.6% of haplotype 3 homozygotes carry the rs2289728 SNV. Additionally, haplotype 4 homozygotes carry the two PD SNVs, rs356182 and rs5019538, at a 74.50% and 73.20% frequency respectively. Table 1 describes the full association and LD results.

**Table 1:** Results of the association and LD between the CT-rich *SNCA* haplotypes and the risk SNVs

Haplotype	Allele Frequency	Reported Frequency	Risk SNP	Phenotype	t	P	SE	Rsq	D'
1	0.26	0.28	rs356182	PD	-9.153	1.65E-19	0.024	0.04	0.45
			rs5019538	PD	-6.663	3.68E-11	0.025	0.03	0.39
			rs2298728	PD	-6.343	2.93E-10	0.046	0.02	1.00
			rs7680557	LBD	-13.007	7.86E-37	0.023	0.08	0.49
2	0.29	0.33	rs356182	PD	-11.372	7.26E-29	0.023	0.09	0.59
			rs5019538	PD	-9.890	2.01E-22	0.023	0.06	0.57
			rs2298728	PD	-5.357	9.68E-08	0.044	0.03	0.91
			rs7680557	LBD	21.539	1.64E-90	0.020	0.22	0.71
3	0.08	0.08	rs356182	PD	7.367	2.78E-13	0.016	0.03	0.43
			rs5019538	PD	-5.250	1.73E-07	0.016	0.01	0.59
			rs2298728	PD	45.483	2.88E-290	0.020	0.55	0.76
			rs7680557	LBD	10.483	6.56E-25	0.015	0.05	0.75
4	0.37	0.31	rs356182	PD	16.417	4.53E-56	0.022	0.15	0.40
			rs5019538	PD	22.485	1.86E-97	0.021	0.24	0.55
			rs2298728	PD	-8.218	5.24E-16	0.044	0.02	0.80
			rs7680557	LBD	-12.963	1.32E-36	0.022	0.09	0.41

*t* T-value, *P* P-value, *SE* Standard Error, *Rsq* R-squared, D’ D Prime.

Despite the modest sample size for an association analysis, we next assessed the relationship between the four haplotypes and risk of PD. After correcting for covariates, there was no significant association between any of the four haplotypes and the PD phenotype at a p-value of 0.05 in our data (see Table 2). However, in the same dataset, the PD risk rs356182 SNV was significantly associated with PD at *p* = 6 × 10^-03^ (OR = 1.05, 95% CI = 0.0147–0.0855) which is in line with the most recent PD meta-analysis (*p* = 3.89 × 10^-154^, OR = 1.32).

**Table 2:** Results of the CT-rich *SNCA* haplotypes’ and risk SNVs’ association with the PD phenotype

Haplotype/SNV	t	P	SE	OR	2.50%	97.50%
Haplotype 1	-0.781	4.35E-01	0.018	0.990	-0.0496	0.0213
Haplotype 2	1.273	2.11E-01	0.019	0.860	-0.0132	0.0621
Haplotype 3	1.252	2.16E-01	0.029	1.040	-0.0202	0.0916
Haplotype 4	-1.254	2.10E-01	0.019	0.980	-0.0602	0.0132
rs356182 (PD)	2.773	5.62E-03	0.018	1.050	0.0147	0.0855
rs5019538 (PD)	1.824	6.84E-02	0.018	1.030	-0.0025	0.0692
rs2298728 (PD)	0.887	3.75E-01	0.034	1.030	-0.0364	0.0967
rs7680557 (LBD)	0.772	4.41E-01	0.017	1.010	-0.0208	0.0478

*t* T-value, *P* P-value, *SE* Standard Error, *OR* Odds Ratio.

### Different haplotypes are associated with alternative SNCA transcript in the brain

**Fig. 1:**
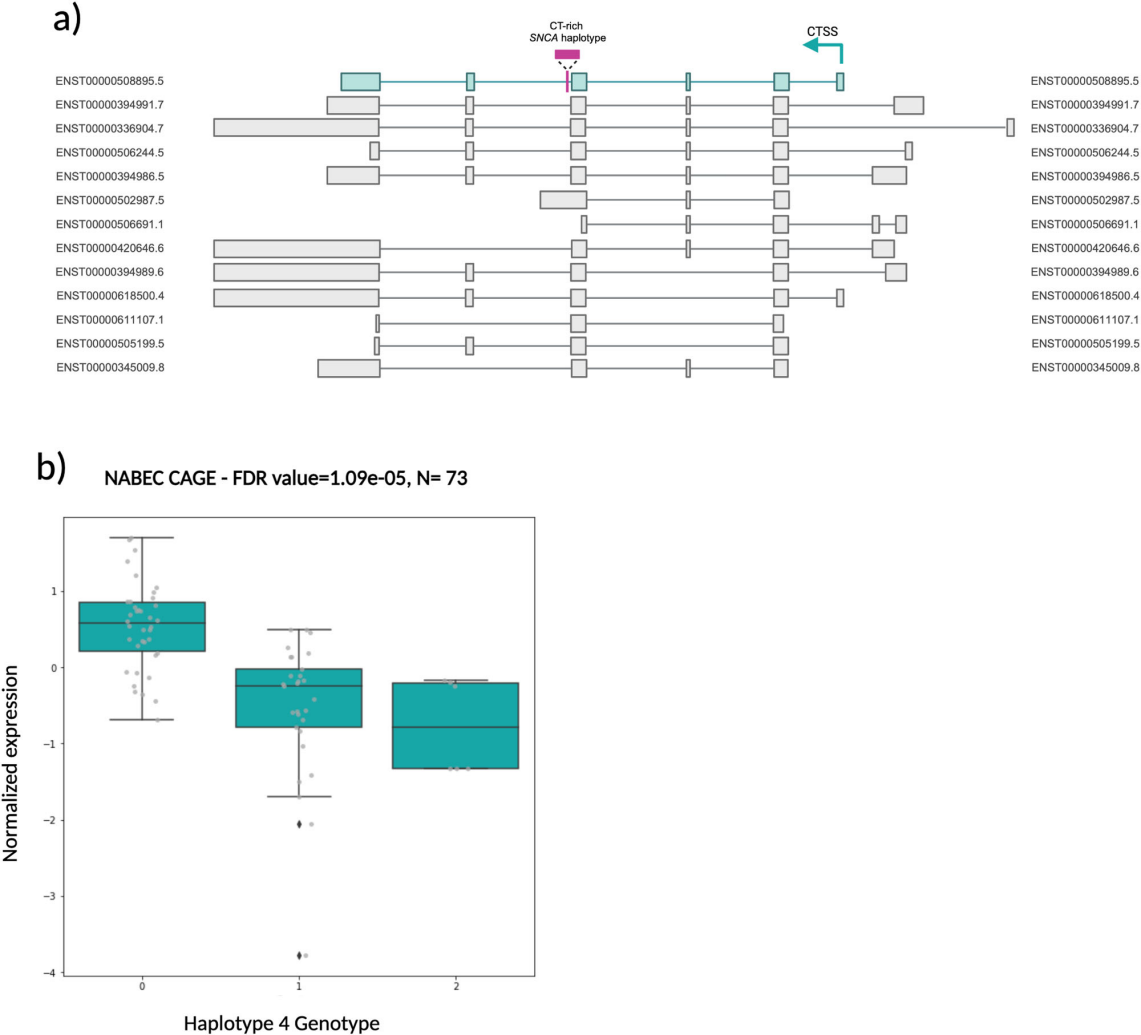
The CT-rich *SNCA* haplotype 4 impacts gene expression in the frontal cortex. **a** Transcript model of *SNCA* with blue-shaded significantly differentially expressed transcript, ENST00000508898.5. Pink box denotes CT-rich *SNCA* haplotype location in intron 4. **b** Box plot for haplotype 4 QTL from NABEC CAGE-seq data for CAGE transcription start site (CTSS) chr4:89836178-89836272. CTSS expression normalized through quantile regularization and changed to normal distribution. Expression of CTSS decreases with presence of haplotype 4. Center line represents the median and error bars indicate the maximum and minimum quartiles.

Next, we assessed if any of the haplotypes had an effect on expression of *SNCA* in the brain. For this, we used short read RNAseq and CAGEseq data generated from the NABEC cohort from brain frontal cortex with matched ONT whole genome long-read sequencing data. After basecalling and mapping of the long-read data, we manually genotyped the NABEC cohort (*n* = 204) on IGV for the four *SNCA* haplotypes. Using these calls and the matched gene level short read RNA-seq data (*n* = 202), QTL analyses were performed and through this, no haplotype was identified as a significant QTL. Additionally, our four risk SNVs were also not significant for gene-level QTL results from the RNA-seq data (Supplementary Table 2). From the PPMI data, rs2298728 was a significant QTL for *SNCA* at the gene level (FDR = 5.09 × 10^-03^) but no other SNV or haplotype was a significant QTL at an FDR < 0.01 (supplementary Table 3). Next, we assessed if there were any differences in transcription start sites usage using CAGEseq data. Long-read ONT NABEC samples with matching CAGEseq data (*n* = 78) were included in the analysis. After filtering, we identified 37,132 different transcription start sites, of which 1583 were located in chromosome four and included in the analysis. Haplotype 4, harboring the large insertion, was called as a significant QTL (FDR = 3.76 × 10^-8^) (Table 3) at chr4:89836178-89836272, which corresponds to the transcription start site of ENST00000508895.5 and ENST00000618500.4. Haplotype 2 was also called as a significant QTL at this locus (FDR = 5.74 × 10^-6^) although with an inverse beta from haplotype 4. When looking at our risk SNVs, we saw no significance at this TSS for the three PD (rs356182 and, rs5019538, rs2289728) SNVs (FDR = 2.53 × 10^-1^, FDR = 4.89 × 10^-2^, FDR = 6.10 × 10^-2^). However, the LBD (rs7680557) risk SNV was significant at this TSS (FDR = 4.49 × 10^-12^). We additionally saw another SNV in *SNCA* associated with REM sleep behavior, rs3756059, be significant at this TSS (FDR = 1.79 × 10^-12^) (Table 3, Fig. 1).

**Table 3:** Transcriptomic results of the CT-rich *SNCA* haplotypes using CAGE sequencing and bulk gene-level RNA sequencing from brain frontal cortex from the NABEC cohort

Expression Dataset	Cohort	N	Variant ID	AF	P-nominal	FDR	Beta	SE
CAGE (chr4:89836178-89836272)	NABEC	78	Haplotype 1	0.218	3.91E-02	2.71E-01	-0.344	0.164
			Haplotype 2	0.397	5.74E-06	3.52E-04	0.594	0.122
			Haplotype 3	0.115	1.66E-03	4.89E-02	0.700	0.215
			Haplotype 4	0.269	3.76E-08	3.07E-06	-0.792	0.130
			rs3756059	0.463	7.31E-15	1.79E-12	-0.878	0.091
			rs356182 (PD)	0.356	2.98E-02	2.53E-01	-0.324	0.146
			rs5019538 (PD)	0.294	1.80E-03	4.89E-02	-0.465	0.144
			rs2289728 (PD)	0.113	2.49E-03	6.10E-02	0.672	0.215
			rs7680557 (LBD)	0.519	3.67E-14	4.49E-12	0.868	0.094

*AF* Allele Frequency, *P*-nominal P-value nominal, *FDR* False Discovery Rate, *SE* Standard Error.

## Discussion

Here, we describe a large-scale attempt to resolve the *SNCA* CT-rich haplotype, and assess its association with risk of PD. After targeted PacBio sequencing of 1375 cases and 959 controls, we were able to assign each of our samples to one of the four previously reported CT-rich haplotypes. Additionally, the frequencies were in line with those previously reported, which is of note as this is a larger cohort and the first PD cohort to be investigated for these haplotypes.

Through gene expression analysis studies we identified that haplotype 4 (the haplotype associated with PD risk) is a significant QTL in the CAGE-seq from post-mortem frontal cortex brain tissue. In the CAGE-seq data, haplotype 4 is a QTL at the chr4:89836178-89836272 CTSS. This CTSS corresponds to two different *SNCA* transcripts (ENST00000508895.5 and ENST00000618500.4). GTEx data reports that ENST00000618500.4 is not expressed in brain tissue, indicating that this association most likely arises from expression of ENST00000508895.5. The ENST00000508895.5 *SNCA* transcript results in the canonical *SNCA140* isoform which is responsible for approximately 96% of *SNCA* mRNA abundance under normal conditions^14^. Increased expression of *SNCA140* has been shown to result in alpha-synuclein protein aggregation, leading to Lewy Bodies in the brain, a pathological hallmark of PD^15^. In our data, however, haplotype 4 reduces CTSS usage of *SNCA140* which could indicate an alternative mechanism of action. Interestingly, this haplotype was not a QTL on the gene level in the matched short-read bulk RNA-seq. Additionally, 5’ UTR usage has been suggested as an important positive regulator of *SNCA*, and while this is not explored here, UTR usage would be a good target for follow up experiments^16^. Complex SVs such as the one within haplotype 4 are known to contribute to transcript diversity in the human brain, however short-read sequencing methods are limited in their ability to accurately resolve and quantify such transcripts. Hence, further studies into the functional impact of this region would benefit from long-read RNA sequencing to fully elucidate the role of this haplotype in *SNCA* transcript expression.

Examining the relationship of these CT-rich haplotypes with lead disease-linked SNVs identified through GWAS reveals that the LD structure of this region is complex. Here, 90% of homozygous carriers of haplotypes 2 and 3 also carried the LBD *SNCA* SNV (rs7680557), in line with previous reports that haplotypes 2 and 3 were associated with risk of Alzheimer’s Disease with Lewy Bodies. We also confirmed the previously reported strong association between haplotype 3 and the PD risk SNV rs2289728, with 97% of haplotype 3 homozygotes carrying the SNV in at least one allele. However, we were not able to validate the association between rs2289728 and increased *SNCA* expression. Additionally, we identified that 74% of homozygous haplotype 4 carriers also carried two PD *SNCA* risk SNVs (rs356182:A and rs5019538:A) suggesting a complex LD pattern for these PD risk alleles. Our examination of the relationship of each of the risk SNVs and the CT-haplotypes in this dataset failed to reveal a significant association between PD risk and any of the CT-haplotypes, despite being in moderately high LD with known GWAS lead SNVs. While this lack of association may be driven in part by power, it is notable that the variant rs356182 is significantly associated with PD in the current data set, a result that would suggest that at least this well-established GWAS signal is likely not being biologically driven by the CT-rich repeat. This is in line with a recent study of the *SNCA* locus in PD that used large-scale SNV data from over thirty thousand individuals, the results of which the authors interpreted to support the idea that the rs356182 PD risk variant drives the top association signal at *SNCA*^17^.

The CT-rich *SNCA* region cannot be genotyped using short-read methods, hence one potential limitation of this current study arises from the challenges associated with generating long-read sequencing data, primarily due to cost and technical complexity. While the sample size of 1,375 cases and 959 controls is large by long-read standards, it is modest for running association analyses. To increase the statistical power one potential approach would be to impute the genotypes into a much larger case and control cohort. However, despite looking within a 150 kb window, due to the complicated LD structure at this region, we were unable to identify any clear tagging SNVs for the four haplotypes, meaning imputation is currently not possible.

In conclusion, despite its complexity, the *SNCA* genomic region and its regulation is clearly associated with neurodegenerative diseases on several levels. It remains unclear what the exact mechanism is of increased risk for disease in this region. Although the SV is of interest, and our data are limited in terms of sample size, these data do not support a role for the CT-rich region as the primary functional genetic risk factor in that region for PD, suggesting it is more likely that the previously identified SNVs are driving this signal. More ancestry diverse genetic data and additional functional work is needed to fully dissect this region and identify the causal variants for increased risk for PD.

## Methods

### Cohort information

Samples for the targeted Pacbio long read DNA sequencing and RNA QTL were obtained from the Parkinson’s Disease Progression Markers Initiative (PPMI) (https://www.ppmi-info.org/) and the Coriell Institute for Medical Research (https://www.coriell.org/). The study was approved by the institutional review board at each site, and participants provided written informed consent. We sequenced DNA for 894 unrelated PPMI samples (629 PD cases, 265 healthy controls) and 1440 unrelated Coriell samples (746 PD cases and 694 healthy controls). For PPMI PD cases, the age at baseline ranged from 32 to 85 years (mean 61.7 ± 9.8) and for controls the age at baseline ranged from 31 to 84 years (mean 60.3 ± 10.5). For Coriell, PD cases had an age at onset range of 42 to 98 years (mean 68.8 ± 6.2) and healthy controls had an age at baseline range of 61 to 101 years (mean 71.6 ± 7.3). Individuals were not age or gender matched.

**Fig. 2:**
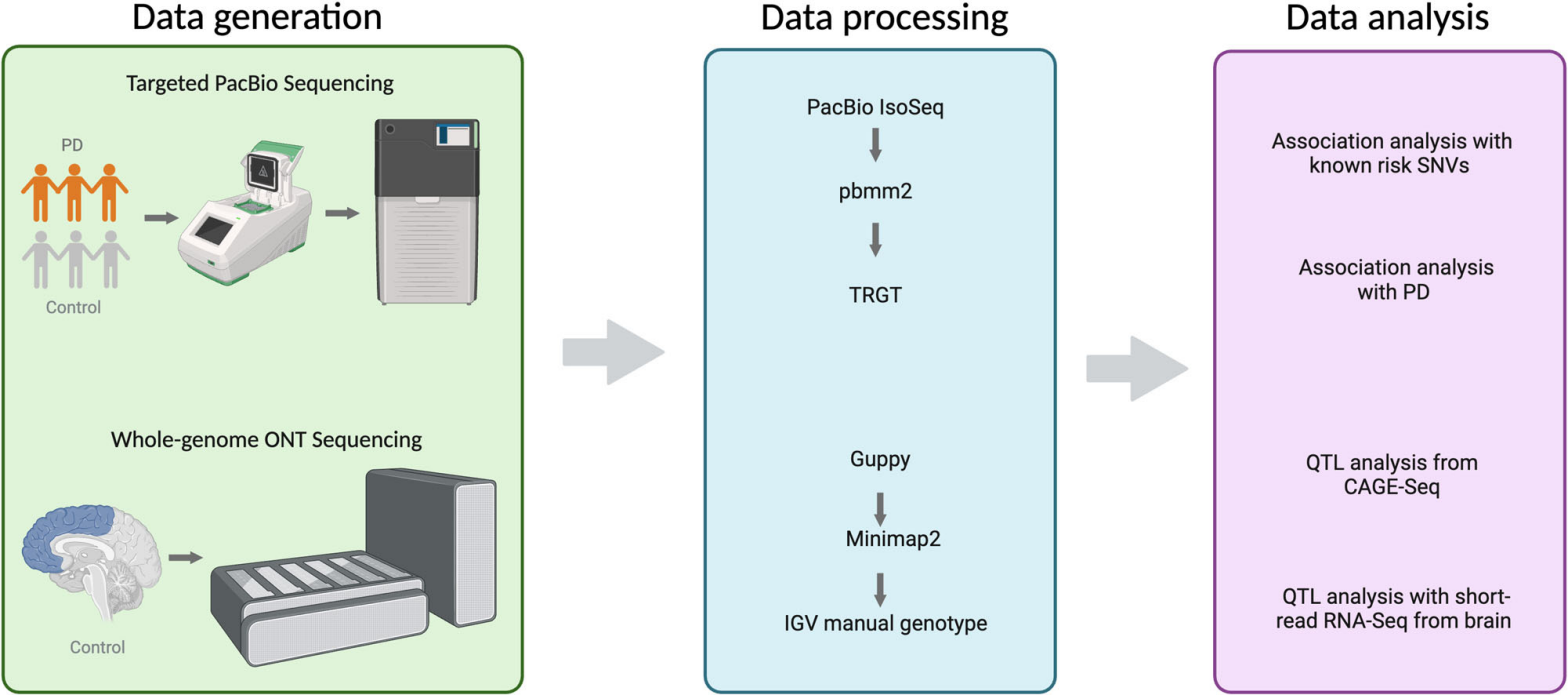
Workflow and rationale summary. Description of study design and rationale behind the analysis included in the work. Created with BioRender.com.

For the downstream QTL analyses, samples were obtained from the North American Brain Expression Consortium cohort^18,19^ for ONT whole-genome long-read DNA sequencing, which contained 204 control samples ranging from 15 to 96 years of age (mean 50.08 ± 26.7). For further cohort demographics, see supplementary table 4. The NABEC study was originally approved by the Joint Addiction, Aging, and Mental Health Data Access Committee and more information can be found at the dbGaP website under the study accession ID phs001300.v4.p1. Out of the 204 samples with ONT long-read data, 202 had matched short-read RNA sequencing data and 78 had matched CAGE sequencing data (Fig. 2).

### Targeted PacBio Long-read DNA Sequencing

To select our region of interest for the amplicon sequencing, we followed PCR guidelines from previous literature of this region^13^. The 520 bp region spanning the CT-rich haplotype in intron 4 of *SNCA* was targeted as follows; 1) the 520 bp region was amplified using RedTaq polymerase with the forward ‘GTTGGAAACTCTCCCAGACACT’ and reverse ‘CAAGCATACCCTTGCCCTGA’ primers. 2) The product of the first PCR was reamplified with the KAPA HiFi HotStart DNA Polymerase and the forward ‘GCAGTCGAACATGTAGCTGACTCAGGTCACGTTGGAAACTCTCCCAGACAC’ and reverse ‘TGGATCACTTGTGCAAGCATCACATCGTAGCAAGCATACCCTTGCCCTGA’ primers. These primers contain universal sequence overhangs at each end. 3) The second PCR product was amplified a third time with unique barcoding primers per sample.

The final amplified and barcoded products were then pooled with equal volume per sample. The pool underwent an AmpurePB (Pacbio) bead clean up at 1.8x ratio and 80% ethanol washes. Each pool contained between 450-490 barcoded samples for a total of five pools. See supplementary table 5 for more details on the PCR protocols.

The “SMRTbell Express Template Prep Kit 2.0” was used to add Pacific Biosciences adapters to 1000 ng of the amplicon pool. The resulting library size and purity was assessed using a Bioanalyzer (Agilent). The primary fragment size was approximately 630-690 bp. The library was run on version 8 M SMRTCells using version 2.0 sequencing reagents. Sequencing was performed at the NIH Intramural Sequencing Center (NISC) on a Sequel II sequencer (Pacific Biosciences) running instrument control software version 11.0.0.144466 and with a 0.2 hour pre-extension and 10 hour movie collection time per SMRTCell. CCS/HiFi reads were generated from the initial subread data from each SMRTCell using the pb_ccs workflow (ccs version 6.3.0) within PacBio SMRTLink version 11.0.0.146107.

### Targeted PacBio Long-read Data analysis

The PacBio IsoSeq methods were then utilized to mark CCS reads with proper forward and reverse primer pairs and chimeric reads were removed. LIMA (version 2.5.1), the PacBio SMRTLink demultiplexing program, was run on the CCS reads using the --isoseq parameter and with a forward-reverse barcode primer set to mark the presence of forward and reverse primers. IsoSeq Refine (isoseq3 version 3.5.0) was then run on the CCS-LIMA-marked reads to find full-length non-chimeric reads by classifying the reads by the presence of 5’ and 3’ primer sequence on ends and barcode sequence orientation and subsequently retaining only reads with barcode with the correct 5’ and 3’ combination. Isoseq Refine generates output.bam and bam.pbi (PacBio-specific index) files and fastqs were then created from the CCS-LIMA Refine bam files. For each sample, pbmm2 (version 1.8.0, PacBio’s implementation of minimap2) was used to run an alignment of the correct refine primer-pair CCS fastq reads against hg38 using parameters: align --preset CCS --sort -j 64. Finally, aligned bam files for samples run over multiple flow cells were merged using samtools (version 1.11).

Sequences for the targeted region were called using TRGT: Tandem Repeat Genotyper (v3.3)^20^ using a chr4:89821175-89821400 (hg38) flank region. Each allele length was then extracted from TRGT’s output VCF using bcftools (v1.17). Any sample with mean depth <10 was removed from the analysis and only samples with European ancestry from HapMap3 calls were retained. The samples were binned into four groups based on allele length following the original haplotype size. For exact allele length grouping within the flank region please see supplementary table 6. Allele frequencies were calculated for each bin and matched against reported variant frequencies. We further confirmed accuracy of our bins by performing an edit distance analysis against original reported sequences.

### Oxford Nanopore Technologies (ONT) Long-read DNA Sequencing

In order to explore potential functional effects of the SV on *SNCA*, we used ONT whole-genome long-read sequencing data from the NABEC cohort^21^ with matched Cap Analysis of Gene Expression (CAGE) and RNAseq data. The protocol for generation of long-read sequencing data from brain frontal cortex tissue is explained in detail in Billingsley et al., 2022^22^. In brief, ~40 mg of frontal cortex frozen tissue was cut and high molecular weight DNA was extracted using a Kingfisher Apex (Thermo Fisher) with PacBio’s Nanobind Tissue Big DNA Kit. The extracted DNA was then sheared to a target size of 30 kb using a Megaruptor 3 (Diagenode). DNA concentration was assessed using the dsDNA BR assay on a Qubit fluorometer (Thermo Fisher) and length was assessed on the TapeStation 4200 (Agilent). Sequencing libraries were prepared with the SQK-LSK 110 kit (ONT) and 400 ng of prepared library per sample was loaded onto R9.4.1 flow cells on ONT’s PromethION device. Samples were sequenced over 72 hours with 1-2 additional library loads per flow cell. Sequencing data resulted in an average coverage of 30x and an N50 of around 30 kb per sample.

### Oxford Nanopore Technologies (ONT) Long-read Data analysis

ONT long-read sequencing data was basecalled using Guppy/6.1.2 and mapped with minimap2/2.24^23^. Because TRGT can only analyze PacBio data, we manually genotyped the NABEC cohort through visualization on the Integrative Genome Browser (IGV)^24^.

### NABEC CAGE sequencing

Generation of CAGE sequencing data was previously described in Blauwendraat et al.^25^. In brief, total RNA was extracted from the frontal lobe of the human brain. The sequencing library was prepared according to a standard method. Strand-specific sequencing was performed using the Illumina HiSeq 2000 for 50 bp single end reads. CAGE seq data was analyzed by nf-core/cage-seq^26^ using default parameters. The pipeline performed quality control of raw reads by FastQC^27^, trimming reads by Cutadapt^28^, read alignment by STAR^29^, CAGE tag counting and clustering by paracle^30^, CAGE tag clustering QC by RSeQC^31^, and quality control of all results by MultiQC^27^. The clustered CAGE-defined transcription start sites (CTSS) counted numbers from nf-core/cage-seq (count_table.tsv) were filtered using the following rules; (1) CAGE clusters identified in more than 10 samples with more than 5 reads (2) CAGE clusters identified in 90% of the samples (3) the age of the sample is larger than 15. Using the filtered CTSS counted numbers, we performed QTL analysis.

### Genetic analysis

Genotypes for known *SNCA* risk SNVs for PD and Lewy Body Dementia (LBD) from the most recent GWAS^2,32^ were extracted for our samples using PLINK/1.9^33^ from the AMP-PD/GP2 variant calls (rs658182, rs5019538, and rs2298728 for PD, rs7680557 for LBD). Haplotypes were coded as 0/1/2 based on the genotype and we then ran a linear regression in R between each haplotype bin and risk SNV using sex, age, and PCs 1–5 as covariates. A generalized linear model (GLM) was run between each haplotype group and the PD phenotype using sex, age, and PCs 1-5 as covariates. These analyses were run on each cohort separately and jointly.

### NABEC Bulk short-read RNA seq

To consider expression in the frontal cortex, sample data was taken from the North American Brain Expression Consortium (NABEC; dbGaP Accession phs001300.v1.p1). Bulk RNA-seq data from all samples was quantified using Salmon^34^ with the filtered Gencode v32 human transcriptome index. Bulk frontal cortex expression from NABEC contained 202 samples covering 60,177 quantified genes. Sex specific genes were subsetted and plotted to identify any sample gender mismatches, and for brain samples, donors under 15 years old were also removed. 32,307 genes with a missingness below 0.33 were excluded, leaving 17,293 well detected genes to continue through normalization. After removing low variance (4330) the remaining 12,963 genes were plotted via UMAP (v0.5.3) and their coordinates used as covariates to correct 17,293 well detected genes.

### PPMI Short-read RNA-sequencing

We obtained whole blood RNAseq data from PPMI at baseline to assess if gene expression was correlated with any of the SNCA haplotypes. Whole blood RNAseq generation is described in detail by Van Keuren-Jensen et al.^35^. In brief, PAXgene blood miRNA kit (Qiagen) was used to isolate total RNA, followed by DNase treatment. DNase-treated RNA samples were sent to the HudsonAlpha Institute of Biotechnology (HAIB) for library preparation and sequencing. Stranded libraries were prepared using the New England Biolabs (NEB) Ultra II First Strand Module (catalog number E7771L) followed by the NEB Ultra II Directional Second Strand Module (catalog number E7550L). cDNA was converted by standard, ligation-based library preparation and each library amplified for 12 cycles of PCR. A unique index sequence was used on each forward and reverse primer. Libraries were diluted to 2 nM final stocks, pooled in equal molar amounts, sequenced on the Illumina NovaSeq 6000 platform, and demultiplexed based on the unique indexes. STAR v2.6.1d^29^ was used to align FASTQs to the human genome (B38). Gene counts were generated with featureCounts v1.6.2^36^ using the GENCODE29 annotation. Transcripts were estimated with salmon v0.11.3^34^ from fastq files using GENCODE298.

### QTL analysis

We used the gene expression data of gene-level as an input and excluded the gene of which the TPM is smaller than one in more than one-third of the subjects. The expression data was normalized by quantile-normalization. UMAP was calculated from the normalized expression data and we then used UMAP to adjust the expression data. Expression data with quantile normalized and UMAP adjusted data was used for cis expression quantitative trait loci analysis using tensorQTL^37^. Genetic PC 1-5, sex, age were used as covariates. All variant–phenotype pairs within a specified window (±1 Mb) around the phenotype were calculated.

### Supplementary information

Supplementary Material

## Data Availability

For the PPMI cohort, the short-read *SNCA* SV genotypes are available at the LONI IDA. For the NABEC cohort, the long-read *SNCA* SV genotypes are available from dbGaP, study accession phs002636.v1.p1.

## Code availability

Analysis pipeline is publicly available at https://github.com/neurogenetics/SNCA_intron4_SV/blob/main/README.md.
