# Supplementary material for: Characterizing a complex CT-rich haplotype in intron 4 of SNCA using large-scale targeted amplicon long-read sequencing
Source: NPJ Parkinsons Dis. 2024 Jul 26;10:136. doi: 10.1038/s41531-024-00749-4 (PMC11282088; doi:10.1038/s41531-024-00749-4)
Supplement: Supplementary file 1 — Supplementary Material [file 41531_2024_749_MOESM1_ESM.pdf]

Haplotype	Frequency PPMI (n=725)	Frequency Coriell (n=1118)	Frequency All (n=1842)	Reported Frequency
1	23%	29%	27%	28%
2	40%	21%	28%	33%
3	6%	10%	8%	8%
4	31%	40%	37%	31%

Supplementary table 1: Allele frequencies for each CT-rich SNCA haplotype split up by cohort.

Expression Dataset	Cohort	N	Variant ID	AF	P-nominal	FDR	Slope	Slope SE
Bulk RNA (*SNCA*)	NABEC	202	Haplotype 1	0.205	5.60E-01	9.05E-01	-0.057	0.098
			Haplotype 2	0.418	2.45E-01	7.44E-01	0.094	0.081
			Haplotype 3	0.094	4.54E-01	9.05E-01	0.114	0.152
			Haplotype 4	0.282	1.98E-01	7.44E-01	-0.124	0.096
			rs356182 (PD)	0.349	6.24E-01	9.05E-01	-0.042	0.086
			rs5019538 (PD)	0.291	8.52E-01	9.33E-01	-0.017	0.092
			rs2289728 (PD)	0.094	5.68E-01	9.05E-01	0.086	0.150
			rs7680557 (LBD)	0.500	2.26E-01	7.44E-01	0.099	0.082

Supplementary table 2: NABEC RNA-seq QTL results. AF= haplotype allele frequency, P-nominal = nominal p-value, FDR= false discovery rate, SE= standard error

Expression Dataset	Cohort	Variant ID	AF	P-nominal	FDR	Slope	Slope SE
Bulk RNA (*SNCA*)	PPMI	Haplotype 1	0.225	3.07E-03	1.23E-02	0.201	0.068
		Haplotype 2	0.401	3.09E-02	4.95E-02	-0.127	0.059
		Haplotype 3	0.052	1.36E-02	3.62E-02	0.301	0.122
		Haplotype 4	0.323	5.46E-02	7.28E-02	-0.137	0.071
		rs356182 (PD)	0.384	4.65E-01	4.65E-01	-0.042	0.058
		rs5019538 (PD)	0.324	2.35E-02	4.70E-02	-0.132	0.058
		rs2298728 (PD)	0.059	6.36E-04	5.09E-03	0.399	0.116
		rs7680557 (LBD)	0.490	3.35E-01	3.82E-01	-0.053	0.055

Supplementary table 3: PPMI RNA-seq QTL results. AF= haplotype allele frequency, P-nominal = nominal p-value, FDR= false discovery rate, SE= standard error

Study	Cases (N)	Controls (N)	Total (N)	Female case (%)	Female control (%)	Case age at baseline/onset (mean , SD in years)	Control age at baseline (mean, SD in years)
**PPMI**	629	265	894	40.00	42.00	61.7, 9.83	60.3, 10.5
**Coriell**	746	694	1440	48.00	50.00	68.8, 6.2	71.6, 7.3
**NABEC**	NA	204	204	NA	35.00	NA	40.08, 26.65

Supplementary table 4: Clinical and demographic characteristics of samples included in long-read and transcriptomic analyses. SD: standard deviation; NA: Not available

PCR1

Reagent	Volume
H2O	5µL
REDTaq Readymix PCR Reaction Mix	12.5µL
FW Primer (5µM)<br>5’ GTTGGAAACTCTCCCAGACACT 3’	2.5µL
RV Primer (5µM)<br>5’ CAAGCATACCCTTGCCCTGA 3’	2.5 µL
Control DNA (10ng/µL)	2.5µL

Protocol
94.0 °C, 3:00 minutes
94.0 °C, 1:00 minutes (x30)
55.1 °C, 0:30 minutes (x30)
72.0 °C, 1:00 minutes (x30)
72.0 °C, 10:00 minutes

PCR2

Reagent	Volume
H2O	16µL
KAPA HIFI	5µL
dNTPs	0.75µL
FW Primer (10µM)<br>5'<br>GCAGTCGAACATGTAGCTGACTCAGGTCACGTTGGAAACTCTCCCAGACAC<br>3'	0.75µL
RV Primer (10µM)<br>5'<br>TGGATCACTTGTGCAAGCATCACATCGTAGCAAGCATACCCTTGCCCTGA<br>3'	0.75µL
Polymerase	0.75 µL
PCR1 Product (1:200 dilution)	1µL

**Protocol**
95.0 °C, 0:30 minutes (x10)
57 °C, 0:30 minutes (x10)
72.0 °C, 1:00 minutes (x10)

PCR3

Reagent	Volume
H_2O	14µL
KAPA HIFI	5µL
dNTPs	0.75µL
1 uM Unique Barcoding Primers (FW+RV)	3.75µL
Polymerase	0.5 µL
PCR2 Product (1:20 dilution)	1µL

Protocol
95.0 °C, 0:30 minutes (x15)
57 °C, 0:30 minutes (x15)
72.0 °C, 1:00 minutes (x15)

Supplementary table 5: PCR CT-rich SNCA amplification protocol description

	Flank Allele Length (bp)	
**Haplotype**	**Greater Than**	**Less Than**
1	223	227
2	NA	224
3	226	291
4	290	NA

Supplementary table 6: TRGT flanking bins

Haplotype 1

3' TACTTTTTTTCCTTCTTTCTTTCTTTTTCTTTTTCTTTCTTTCTTTCCTTCTTTCTCTCTTTCCTTTCTTTCTTTTCCCT
TCCTTCCTTCCTTTCTCCCTTCCTTCCTTCCTCCCTCTCTCCCTCCCTTCCTTCCTCCCTTTCTTTCTTTCTCTTTTTTCTTTCTTGCTTCCTTCCTTCCTTCTTT
CCTTTTCTTTCTTTTTCCTTTCTTTGCCAAAGTGTTATTC 5'

Haplotype 2

3'TACTTTTTTTCCTTCTTTCTTTCTTTTTCTTTTTCTTTCTTTCTTTCCTTCTTTCTCTCTTTCCTTTCTTTCTTT**A**C**T**~~C~~
TTC**T**TTCCTTT**T**TTTTCT**TT**~~C~~T**C**CCTTCCTTCCTCCCTCTCTCCCTCCCTTCCTTCCTCCCTTTCTTTCTTTCTCTTTTTTCTTTCTTGCTTCCTTCCTTCCTTCTT
TCCTTTTCTTTCTTTTTCCTTTCTTTGCCAAAGTGTTATTC 5'

Haplotype 3

3' TACTTTTTTTCCTTCTTTCTTTCTTTTTCTTTTTCTTTCTTTCTTTCCTTCTTTCTCTCTTT[23bp]CCTTTCTTTCTT
TTCCCTTCCTTCCTTCCTTTCTCCCTTCCTTCCTTCCTCCCTCTCTCCCTCCCTTCCTTCCTCCCTTTCTTTCTTTCTCTTTTTTCTTTCTTGCTTCCTTCCTTC
CTTCTTTCCTTTTCTTTCTTTTTCCTTTCTTTGCCAAAGTGTTATTC 5'

Haplotype 4

3' TACTTTTTTTCCTTCTTTCTTTCTTTTTCTTTTTCTTTCTTTCTTTCCTTCTTTCTCTCTTT**T**CTTTCTTTCTTT**A**C
[95bp]CCTTCCTTC**T**TTCCTTTCT**TT**CTT[3bp]CCTTCCTTCCTCCCTCTCTCCCTCCCTTCCTTCCTCCCTTTCTTTCTTTCTCTTTTTTCTTTCTTGCTTCCT
TCCTTCCTTCTTTCCTTTTCTTTCTTTTTCCTTTCTTTGCCAAAGTGTTATTC 5'

Supplementary Figure 1: Sequences for the CT-rich *SNCA* haplotype. Haplotype 1 is the reference sequence corresponding to chr4:89821175-89821400 on the minus strand.
